# Literary Notes

**Published:** 1908-12

**Authors:** 


### LITERARY NOTES.

An English-Chinese lexicon of medical terms, prepared by Dr. Philip B. Cousland, has just been published in Shanghai. Though the author is an Englishman by birth, he has based his book largely upon the Medical Dictionary of Dr. George M. Gould, formerly of Philadelphia, now of Ithaca, which may be considered a compliment to American scholarship. Dr. Cousland has recently published a translation of Professor Halliburton's edition of Kirkes' Physiology.

William Wood & Company, Publishers, New York, announce the publication of new books entitled: Common Affections of the Liver by W. Hale White, M.D., F.R.C.P. 12 mo, 310 pages. Price, muslin, $2.00 net. The Bacteriology of the Eye by Dr.

Theodor Axenfeld, Professor of Ophthalmology in the University of Freiburg. Translated by Angus Macnab, B.A., B.Sc., M.B., Ch.B., F. R. C. S. Price, $6.00 net. The Ophthalmic and Cutaneous Diagnosis of Tuberculosis, together with a Discussion of the Clinical Methods for the Early Diagnosis of Pulmonary Tuberculois by Dr. Alfred Wolff-Eisner. A Preface by Professor H. Sevator and an Introductory Note to the English reader by C. Theodore Williams, M.D. (Translated from the German by Bernard I. Robert.) Octavo, 215 pages, illustrated by numerous diagrams and large folding charts and two chromo-lithographic plates. Price, extra muslin, $2.75 net.

